# Discrimination, Stress, and Health Across the Life Course

**DOI:** 10.1093/geroni/igaa057.1933

**Published:** 2020-12-16

**Authors:** Roland Thorpe, Carl Hill

## Abstract

There is a paucity of research that seeks to understand why race disparities in health across the life course remain elusive. Two such explanations that have been garnering attention is stress and discrimination. This symposium contains papers seeking to address the impact of discrimination or stress on African American health or health disparities across the life course. First, Nguyen and colleagues examine 1) the associations between discrimination and objective and subjective social isolation and 2) how these associations vary by age in using data from the National Survey of American Life. Discrimination was positively associated with being subjectively isolated from friends only and family only. This relationship varied by age. Discrimination did not predict objective isolation. Second, Brown examines evidence of the black-white paradox in anxiety and depressive symptoms among older adults using data from 6,019 adults ages 52+ from the 2006 HRS. After adjusting for socioeconomic factors, everyday discrimination, chronic conditions, and chronic stress, there are no black-white differences in anxiety and depressive symptoms. Third, Cobb and colleagues investigate the joint consequences of multiple dimensions of perceived discrimination on mortality risk using mortality data from the 2006-2016 HRS. The authors report the number of attributed reasons for everyday discrimination is a particularly salient risk factor for mortality in later life. This collection of papers provides insights into how discrimination or stress impacts African American health or health disparities in middle to late life.

